# Robotic Versus Conventional Unicompartmental Knee Surgery: A Comprehensive Systematic Review and Meta-Analysis

**DOI:** 10.7759/cureus.46681

**Published:** 2023-10-08

**Authors:** Ahmed Hussein Ghazal, Zien Alabdin Fozo, Sajeda G Matar, Ibrahim Kamal, Mohamed Hesham Gamal, Khaled M Ragab

**Affiliations:** 1 Orthopaedics, Northwick Park Hospital, London North West University Healthcare NHS Trust, Harrow, GBR; 2 Orthopaedics, Ysbyty Gwynedd Hospital, Bangor, GBR; 3 Pharmacology and Therapeutics, Faculty of Pharmacy, Applied Science Private University, Amman, JOR; 4 General Medicine, Al-Azhar University, Alexandria, EGY; 5 Pharmacology and Therapeutics, Faculty of Pharmacy, Tanta University, Tanta, EGY; 6 Faculty of Medicine, Minia University, Minia, EGY

**Keywords:** unicompartmental knee arthroplasty, robotic-assisted, conventional surgery, uka, meta-analysis

## Abstract

Robotic-assisted surgery is a computer-controlled technique that may improve the accuracy and outcomes of unicompartmental total knee arthroplasty (TKA), a partial knee replacement surgery. The purpose of a meta-analysis about robotic-assisted versus conventional surgery for unicompartmental TKA is to compare the effectiveness of these two methods based on the current evidence. Our meta-analysis can help inform clinical decisions and guidelines for surgeons and patients who are considering unicompartmental TKA as a treatment option. We searched four online databases for studies that compared the two methods until March 2023. We used RevMan software to combine the data from the studies. We calculated the mean difference (MD) and the 95% confidence interval (CI) for each outcome, which are statistical measures of the difference and the uncertainty between the two methods. We included 16 studies in our analysis. We found that robotic-assisted surgery had a better hip-knee-ankle angle, which is a measure of how well the knee is aligned, than conventional surgery (MD = 0.86, 95% CI = 0.16-1.56). We also found that robotic-assisted surgery had a better Oxford Knee score, which is a measure of how well the knee functions, than conventional surgery (MD = 3.03, 95% CI = 0.96-5.110). This study compared the results of conventional and robotic-assisted unicompartmental knee arthroplasty in 12 studies. We concluded that robotic-assisted surgery may have some benefits over conventional surgery in terms of alignment and function of the knee. However, we did not find any significant difference between the two methods in terms of other outcomes, such as pain, range of motion, health status, and joint awareness. Therefore, we suggest that more research is needed to confirm these results and evaluate the long-term effects and cost-effectiveness of robotic-assisted surgery.

## Introduction and background

Knee osteoarthritis affects a large population of people. Among people aged 45 years old and above, up to 19% have knee osteoarthritis, while nearly 27 million suffer from osteoarthritis in the United States [1], leading to a low quality of life and persistent pain [2]. According to Nguyen et al., the prevalence of knee pain increased by 65% from 1974 to 1994 in the United States [3], while another study conducted among African Americans and Caucasians indicated that 43% of the population have knee pain [4].

When one part of the knee joint is affected by osteoarthritis, a condition that causes the cartilage to wear away and the bones to rub against each other, a surgical procedure called conventional unicompartmental knee arthroplasty (UKA) can be performed to replace the damaged part with an artificial implant [5]. This procedure has become more popular and widely used because it preserves most of the natural bone and uses small incisions that cause less damage to the surrounding soft tissues, resulting in less blood loss and faster recovery than a total knee replacement surgery that replaces the entire joint [6]. However, this procedure also has some drawbacks, such as a higher risk of failure, especially for patients who have a high body mass index (BMI), which is a measure of body fat based on height and weight [7,8].

Robotic-assisted UKA was first introduced to decrease the rate of malposition of the component and thus, theoretically, to provide better clinical results and survival [9-12]. A study by Negrín et al. indicated that robotic-assisted UKA results in higher precision and lower pain [2].

In this study, we aim to assess the safety and efficacy of robotic-assisted compared to conventional UKA.

## Review

Methodology

Study Design

We followed the Preferred Reporting Items for Systematic Reviews and Meta-Analyses (PRISMA) guidelines in this study. We also adhered to the steps described by the Cochrane Handbook for Systematic Reviews of Interventions [13,14].

Literature Search Strategy

A systematic search was conducted through PubMed, Web of Science, Cochrane Library, and Scopus until February 2023 using the following research strategy: (robot OR robotic OR “robotic surgical procedure” OR “robotic arm assisted”) AND (Arthroplasty OR Replacement) AND Knee. All included articles were in the English language, and the references of the included articles were manually screened to ensure all matched articles were included in this study.

Inclusion and Exclusion Criteria

We selected studies that met the following criteria: randomized controlled trials (RCTs), cohort studies, or case-control studies; studies enrolling patients who had UKA, which is a surgery that replaces only one part of the knee joint; studies that compared the outcomes of robotic-assisted surgery, which uses a computer-controlled device to assist the surgeon, with conventional surgery, which does not use any robotic device; and studies that reported data on the safety or efficacy of the two methods. We did not exclude any studies based on the cause of UKA. We excluded studies that were not relevant to our research question; such as studies that used animals; abstract presentations at conferences; reviews, book chapters, thesis, editorials, letters, or abstract-only papers; and studies that were not written in English.

Data Extraction and Quality Assessment

The search strategy was used by two authors independently to review the literature and select articles that met the inclusion and exclusion criteria. The data from the selected articles were also extracted independently by both authors. We assessed the risk of bias using the Cochrane Risk of Bias tool (version 1), as described in chapter 8.5 of the Cochrane Handbook. This tool evaluates the quality of the studies by examining the potential biases in the selection, performance, detection, attrition, reporting, and other aspects. The risk of bias can be high, low, or unclear for each aspect. We also used the National Institutes of Health (NIH) tool for risk of bias to assess cohort and case-control studies. This tool consists of 12 questions about the population and sample size justification, research question, definition of control, inclusion criteria and cases, time of event, blinding, and confounding factors.

Outcomes of Interest

The following outcomes were used: (a) change in hip-knee-ankle angle; (b) change in International Knee Society (IKS) score [15]; (c) change in Oxford Knee score [16]; (d) change in range of motion; (e) forgotten joint score [17]; (f) hip-knee-ankle angle postoperatively; (g) health status by 12-Item Short Form Health Survey (SF-12) such as physical functioning, and mental health [18]; (h) tibial slope; and (i) Western Ontario and McMaster Universities Osteoarthritis (WOMAC) pain score [19].

Statistical Analysis

We used the Review Manager (RevMan) software version 5.4 to perform the statistical analysis. We set the p-value value <0.5 as the level of significance. We calculated the mean difference (MD) and the 95% confidence interval (95% CI) for the outcomes data, which were continuous. We also tested the heterogeneity of the data using the I^2^ test and the chi-square test. We considered the data to be heterogeneous if the p-value of the chi-square was less than 0.1 and the I^2^ value was more than 50%. We used the fixed-effect model to analyze the data that were homogeneous, and the random-effect model to analyze the data that were heterogeneous.

Results

Literature Search

**Figure 1:**
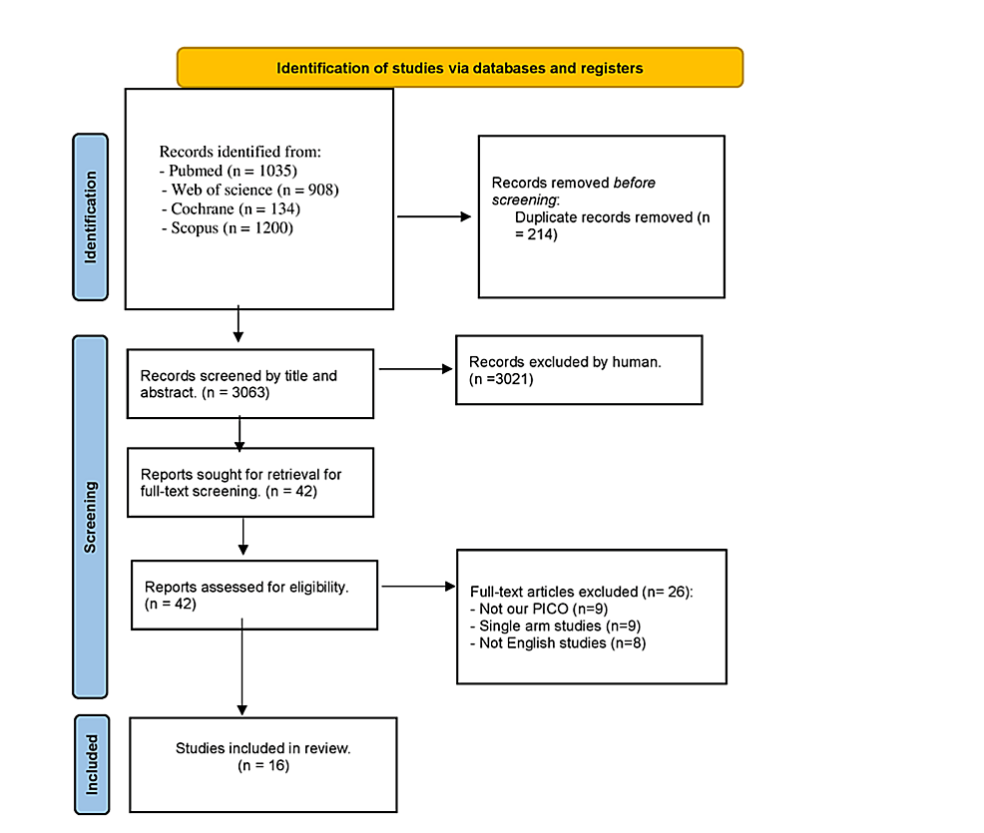
Preferred Reporting Items for Systematic Reviews and Meta-Analyses flow diagram.

Our literature search yielded 3063 records after duplication removal from our databases. In total, 42 articles were included for full-text screen after screening the titles and abstracts. A total of 16 studies [2,20-34] matched our inclusion criteria in the qualitative and quantitative synthesis (Figure 1).

Characteristics of Included Studies

We included 16 studies and their specific details regarding summary and baseline characteristics are presented in Table 1.

**Table 1:** Summary and baseline characteristics of the included studies. UKA: unicompartmental knee arthroplasty; IKS: International Knee Society; FJS: forgotten joint score; SF-12: 12-Item Short Form Health Survey; WOMAC: Western Ontario and McMaster Universities Osteoarthritis

Study ID	Study arms	Study design	Study arms, N	Female, N	Age, mean (SD)	BMI, mean (SD)	Operated side R (L)	Follow-up duration	Inclusion criteria	Primary outcomes	Conclusions
Banger et al. (2020) [34]	Intervention	Randomized controlled trial	38	20	70.5 (7.1)	32.6 (5.8)	16 (22)	1 Month	“Patients were eligible for inclusion if they presented with medial and lateral compartment OA suitable for treatment with a standard unconstrained TKA, with clinically intact cruciate and collateral ligaments”	“Power calculation for the presence of a biphasic knee flexion moment during gait required 36 patients per group, with a 30% loss to follow-up, totaling 96 recruits. Following slow recruitment, this was changed to a 10% loss to follow-up, with permission of the overview groups, leading to a final recruitment target of 80 patients, which was achieved”	“The study found that robotic arm-assisted bi-UKA maintains the anatomy of the knee in all three planes and alters the overall HKAA much less than a mechanically aligned TKA. Although it remains to be seen whether this will translate into improved long-term outcomes, the results offer the exciting prospect of restoring the pre-disease joint anatomy and producing a kinematic performance that is closer to that of the normal knee”
	Control		32	17	68.7(7.8)	31.7 (17)	14 (18)				
Banger et al. (2021) [29]	Intervention	Randomized controlled trial	49	-	-	-	-	5 years	“Inclusion criteria for UKA have been expanded, based on successful outcomes in young patients (8), obese patients (9), patients with the patellofemoral disease (10), and those who are very active (11). These wider indications have not led to increased adoption of the procedure”	“The primary outcome measure (surgical accuracy)”	“The study showed excellent clinical outcomes in both groups with no statistical or clinical differences in the patient-reported outcome measures. The notable difference was the lower reintervention rate at five years for robotic arm-assisted UKA when compared with a manual approach”
	Control		55	-	-	-	-				
Batailler et al. (2019) [23]	Intervention	Case-control study	80	-	68 (10)	25.5 (3.9)	-	1 year	“Patients undergoing a UKA using the robotic-assisted system between 2013 and 2017”	HKA, IKS score, and tibial slope	“This comparative study reported that robotic-assisted UKA had better positioning than conventional UKA, with similar functional outcomes at mid-term. Revision due to implant malposition or limb malalignment was more common after conventional UKA than robotic-assisted UKA. Long-term follow-up of this cohort was suggested to assess both ongoing survivorship and functional outcome”
	Control		80	53	69 (9.6)	26.1 (4.1)	43 (37)				
Batailler et al. (2023) [30]	Intervention	Randomized controlled trial	33	-	67.1 (8.1)	28.3 (5.6)	19 right	6 months	“Inclusion criteria were an indication of a primary medial UKA for medial osteoarthritis”	HKA, FJS, IKS score, and tibial slope	“Medial UKA restored the physiological axis and corrected varus deformity after both image-free robotic-assisted and varus deformity after both image-free robotic-assisted and after medial UKA were comparable between the techniques. There was no significant difference between these two techniques for clinical outcomes at 6 months after medial UKA”
	Control		33	-	65.6 (7.9)	26.4 (3.5)	19 right				
Bell et al. (2016) [21]	Intervention	Randomized controlled trial	69	39	61.7 (7.9)	-	27 (42)	3 months	“Patients undergoing a UKA using the robotic-assisted or conventional surgery”	Femoral sagittal, femoral coronal, and femoral axial	“Robotic-assisted surgical procedures with the use of the MAKO RIO lead to improved accuracy of implant positioning compared with conventional unicompartmental knee arthroplasty surgical techniques”
	Control		70	40	62.5 (6.9)	-	32 (38)				
Canetti et al. (2018) [24]	Intervention	Retrospective cohort study	17	12	59.5 (9.9)	26.3 (3.8)	-	1 year	“Patients with either a robotic-assisted system or conventional technique for UKA, between April 2012 and December 2016”	FJS, UCLA, and IKSS-F	“Robotic-assisted surgery for lateral UKA reduced the time to return to sports at a patient’s pre-symptomatic level. This robotic tool permitted surgeons to be less invasive regarding soft tissues, including quadriceps muscle, extensor mechanism, and bony resection, which may lead to a shorter recovery. The RTS rates were high in both groups. These results can help surgeons inform patients planning for lateral UKA regarding their anticipated postoperative level of activity, especially in young, active patients with high expectations”
	Control		11	9	66.5 (6.8)	24.2 (4.3)	-				
Clement et al. (2020) [32]	Intervention	Prospective cohort study	90	22	67.8 (8.3)	29.7 (4.9)	-	2 years	“Inclusion criteria included isolated medial compartment osteoarthritis (complete radiological joint space loss); preservation joint space in other compartments of the knee joint; a varus deformity of <10° which is correctible; flexion deformity <15°; and a minimum of 90° of knee flexion”	OKS, FJS, and pain VAS	“Patients with isolated medial compartment arthritis had a greater knee-specific functional outcome and generic health with a shorter length of hospital stay after rUKA when compared to mTKA”
	Control		30	6	65.9 (12.0)	30.5 (8.4)	-				
Crizer et al. (2021) [28]	Intervention	Retrospective cohort study	39	17	58 (13)	28.3 (4.06)	-	2 years	“Patients were included in the analysis if they underwent outpatient unilateral medial UKA, and those who underwent lateral UKA”	Pain VAS and KSS	“Robotic-assisted UKA had superior functional outcomes up until 6 months following surgery, with differences equilibrating between the two cohorts by 1-year postoperatively. While the robotic cohort had lower VAS pain scores at 3 weeks postoperatively, mixed-model regression analysis showed this decrease was not attributable to cohort placement. We also noticed no difference in cumulative postoperative opioid prescriptions, although we were unable to determine precise opioid usage. Those who received robotic assistance were more likely to have their expectations met and satisfaction tended to be higher in the robotic cohort as well. Despite these promising early results, further mid- and long-term studies are needed to better assess whether robotic-assisted UKA provides longer-term benefits on clinical functionality, implant durability, and patient satisfaction. Otherwise, if these outcome metrics are not appreciably impacted by using robotic technology, its broader use will only be considered if robotics can be shown to be cost-effective and time-efficient and eliminate instrument tray burden”
	Control		50	21	63 (11)	28.1 (4.45)	-				
Foissey et al. (2023) [26]	Intervention	Retrospective cohort study	159	98	68.3 (8.1)	27 (3.4)	86 (73)	2–11 year	“Patients undergoing a medial UKA for isolated medial femorotibial osteoarthritis (OA) or osteonecrosis of a femoral condyle or reducible deformity with an intact anterior cruciate ligament (ACL)”	HKA and IKS score	“Robotic assistance led to better accuracy compared to the conventional technique regarding tibial implant positioning in the frontal and sagittal plane, postoperative limb alignment, and JL restoration in patients undergoing medial UKA. This was associated with an improved mid-term sur UKA. This was associated with an improved mid-term survival”
	Control		197	108	66.7 (7.7)	27.5 (3.3)	104 (93)				
Gilmour et al. (2018) [22]	Intervention	Randomized controlled trial	54	29	62.6 (7.13)	-	-	2 years	“Patients undergoing UKA for the treatment of medial compartment osteoarthritis (OA). Participants underwent surgery using either robotic-arm-assisted surgery or conventional manual instrumentation”	“The primary outcome measure (surgical accuracy) has previously been reported (16), as has the 1year secondary exploratory analysis (20). The 2-year analysis of the secondary clinical outcome year secondary exploratory analysis (20). The 2-year analysis of the secondary clinical outcome”	“We have demonstrated that at two years post-operatively, robotic arm assisted 288 technology delivers a clinical outcome that is at least equivalent to a widely used UKA implant and 289 may be superior in more active patients. However, the ceiling effect of our outcome measures May 290 makes it difficult to fully identify any difference in the clinical functional outcome. Nonetheless, we 291 have encouraging early results that suggest improved survivorship and lower postoperative pain in 292 patients undergoing robotic arm-assisted surgery. We will continue to follow the trial participants in 293 the future to assess whether long-term revision rates differ between the two groups, as May 294 has major implications for the cost-effectiveness of the technology (21). Our trial is based on 295 relatively small numbers, and we believe a larger multi-center trial using appropriate outcome 296 measures is required. This would provide sufficient power to perform a sub-group analysis to 297 determine which patients may benefit most from robotic arm-assisted UKA”
	Control		58	35	61.8 (7.84)	-	-				
Negrín et al. (2020) [31]	Intervention	Retrospective cohort study	22	-	-	-	-	1 month	“There was no age limit for inclusion criteria”	Slope before surgery, slope after surgery	“Robotic-assisted UKA shows a better rate of joint line restoration due to less femoral component distalization than conventional UKA. No difference was found in the amount of tibial resection between groups in this study”
	Control		40	-	-	-	-				
Negrín et al. (2021) [2]	Intervention	Retrospective cohort study	18	-	61.75 (8.75)	-	8 right	1–2 years	“Patients who underwent medial UKA between April 2017 and March 2019 in a single center”	Length of hospital stay, surgery mesangial femoral angle, KS	“Robotic-assisted UKA with the NAVIO system offers greater accuracy of femoral implant positioning in the sagittal plane, and it is more accurate in achieving clinical and radiological success compared to conventional surgery”
	Control		16	-	67.5 (6.5)	-	10 right				
Ollivier et al. (2016) [20]	Intervention	Randomized controlled trial	30	17	62 (6.5)	28 (2.75)	18 (12)	1 year	“The inclusion criteria were (1) isolated symptomatic medial femorotibial knee arthritis [2]; (2) with varus deformity; (3) age between 50 and 85 years; and (4) acceptance of a new technology protocol (including the delay between MRI and surgery)”	HKA, tibial slope, ROM, surgery time, sagittal femoral angle	“Our observations suggest that PSI may confer small if any, advantage in alignment, pain, or function after UKA. This argument can therefore not be used to justify the extra cost and uncertainty related to this technique”
	Control		30	17	63 (7)	27 (2.75)	18 (12)				
Park et al. (2019) [33]	Intervention	Retrospective cohort study	57	50	64.4 (3.5)	25.9 (3.7)	-	2 years	“Patients undergoing unilateral primary medial UKA at our institution between January and April 2013”	Coronal alignment, WOMAC, AKS knee score, AKS function score	“In summary, robot-assisted UKA has many advantages over conventional UKA, such as its ability to achieve precise implant insertion and reduce radiologic outliers. Although the clinical outcomes of robot-assisted UKA over a short-term follow-up period were not significantly different compared to those of conventional UKA, there has not been a study revealing the clinical superiority of robotic-assisted UKA despite improved radiologic outcomes, as we have done so in the current study. Notably, this study demonstrates that the findings of several Western studies, which reported the excellence of robot-assisted UKA, are also applicable to Asian patients. Therefore, a longer follow-up period is needed to determine whether the improved radiologic accuracy of the components in robotic-assisted UKA will lead to better clinical outcomes and improved long-term survival”
	Control		55	44	64.8 (3.25)	25.5 (2.5)	-				
Wong et al. (2019) [25]	Intervention	Retrospective cohort study	118	74	67.9 (9.5)	28.7 (4.4)	59 (59)	2 years	“All patients undergoing medial UKA by two senior arthroplasty surgeons at our institution between 2003 and 2014 were eligible for inclusion”	SF-12 mental, SF-12 physical, WOMAC	“This study demonstrated no clear advantages to choosing robotic assistance over the conventional technique for performing a fixed-bearing medial UKA. This study also showed that robotic-arm-assisted UKAs are associated with higher early revision rates. Therefore, fellowship-trained arthroplasty surgeons can rely on conventional methods to perform UKA with confidence in their survivorship and functional outcomes. Overall, institutions should warrant caution in the utilization of RAA UKA which carries an increased cost with limited benefit to patients”
	Control		58	28	70.4 (9.7)	28.2 (5.6)	35 (23)				
Wu et al. (2021) [27]	Intervention	Retrospective cohort study	73	52	69.36 (9.14)	-	31 (30)	2 years	“Patients with medial noncompartmental osteoarthritis who had undergone UKA were recruited for this study. Of them, 73 patients had undergone CUKA between March 2001 and June 2016”	WOMAC	“This study demonstrated that RUKA resulted in higher component positioning accuracy than CUKA. However, a longer surgical time and an increase in blood loss were observed in the RUKA group. No significant differences in clinical outcomes were observed between the two groups. Therefore, a follow-up study may be required to determine whether the increased accuracy of component positioning in RUKA improves clinical outcomes”
	Control		85	41	68.52 (9.78)	-	26 (26)				

Quality Assessment Results

**Figure 2:**
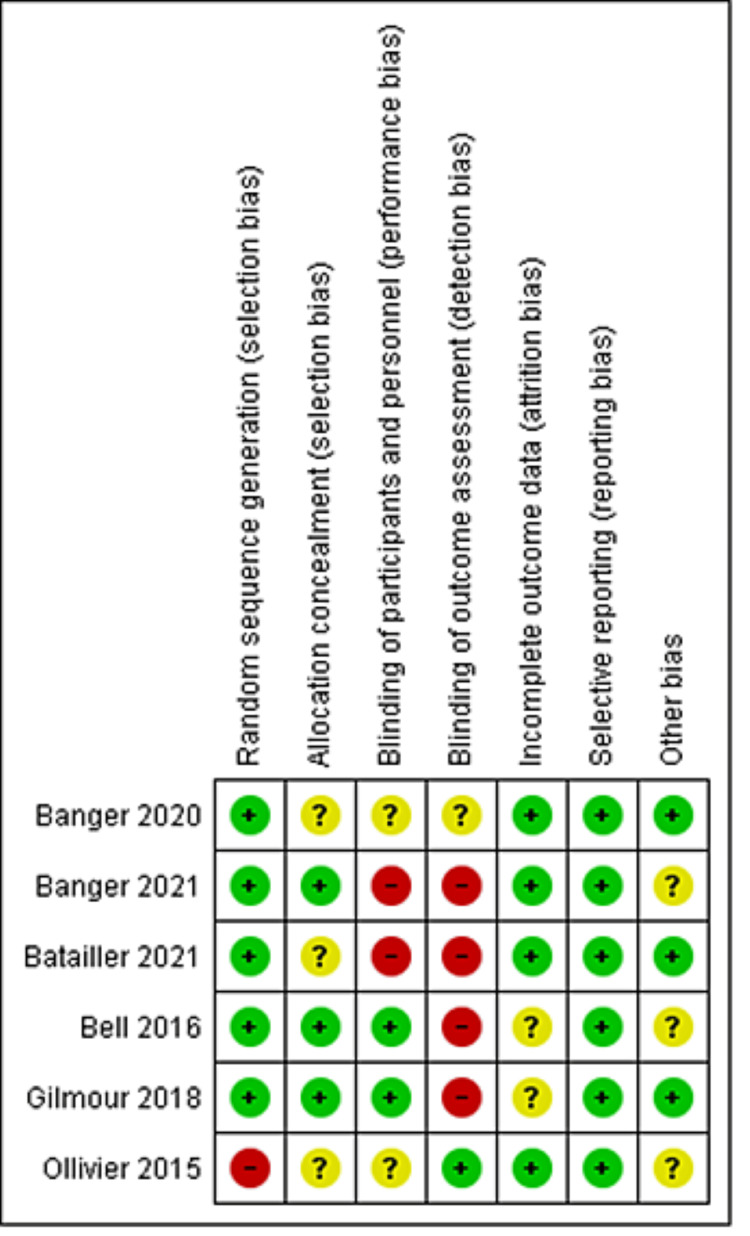
Risk of bias graph summary for randomized controlled trials.

Our included RCTs had a moderate risk of bias, as shown in Figure 2. Regarding cohort and case-control studies, a detailed evaluation is presented in Table 2 and Table 3, respectively.

**Table 2:** NIH quality assessment tool for observational case control studies.

Name	NIH quality assessment tool for observational case-control studies													Quality rating: good (9.5–12 points), fair (6.5–9 points), or poor (6–0 points)
	1. Was the research question or objective in this paper clearly stated and appropriate?	2. Was the study population clearly specified and defined?	3. Did the authors include a sample size justification?	4. Were controls selected or recruited from the same or similar population that gave rise to the cases (including the same timeframe)?	5. Were the definitions, inclusion and exclusion criteria, algorithms or processes used to identify or select cases and controls valid, reliable, and implemented consistently across all study participants?	6. Were the cases clearly defined and differentiated from controls?	7. If less than 100 percent of eligible cases and/or controls were selected for the study, were the cases and/or controls randomly selected from those eligible?	8. Was there use of concurrent controls?	9. Were the investigators able to confirm that the exposure/risk occurred prior to the development of the condition or event that defined a participant as a case?	10. Were the measures of exposure/risk clearly defined, valid, reliable, and implemented consistently (including the same time period) across all study participants?	11. Were the assessors of exposure/risk blinded to the case or control status of participants?	12. Were key potential confounding variables measured and adjusted statistically in the analyses? If matching was used, did the investigators account for matching during the study analysis?	Total scores: Yes = 1/No = 0.5/NR & NA & CD = 0	
	Yes/No/Not reported (NR) or cannot determine (CD) or not applicable (NA)	Yes/No/Not reported (NR) or cannot determine (CD) or not applicable (NA)	Yes/No /Not reported (NR) or cannot determine (CD) or not applicable (NA)	Yes/No/Not reported (NR) or cannot determine (CD) or not applicable (NA)	Yes/No/Not reported (NR) or cannot determine (CD) or not applicable (NA)	Yes/No/Not reported (NR) or cannot determine (CD) or not applicable (NA)	Yes/No/Not reported (NR) or cannot determine (CD) or not applicable (NA)	Yes/No/Not reported (NR) or cannot determine (CD) or not applicable (NA)	Yes/No/Not reported (NR) or cannot determine (CD) or not applicable (NA)	Yes/No/Not reported (NR) or cannot determine (CD) or not applicable (NA)	Yes/No/Not reported (NR) or cannot determine (CD) or not applicable (NA)	Yes/No/Not reported (NR) or cannot determine (CD) or not applicable (NA)		
Batailler et al. (2019) [23]	Yes	Yes	NR	Yes	Yes	Yes	No	Yes	Yes	Yes	NA	NR	8.5	Fair

**Table 3:** NIH quality assessment tool for observational cohort and cross-sectional studies.

Name	NIH quality assessment tool for observational cohort and cross-sectional studies															Quality rating: good (11–14 points), fair (7.5–10.5 points), or poor (0–7 points)
	1. Was the research question or objective in this paper clearly stated?	2. Was the study population clearly specified and defined?	3. Was the participation rate of eligible persons at least 50%?	4. Were all the subjects selected or recruited from the same or similar populations (including the same time period)? Were inclusion and exclusion criteria for being in the study prespecified and applied uniformly to all participants?	5. Was a sample size justification, power description, or variance and effect estimates provided?	6. For the analyses in this paper, were the exposure(s) of interest measured prior to the outcome(s) being measured?	7. Was the time frame sufficient so that one could reasonably expect to see an association between exposure and outcome if it existed?	8. For exposures that can vary in amount or level, did the study examine different levels of the exposure as related to the outcome (eg, categories of exposure, or exposure measured as continuous variable)?	9. Were the exposure measures (independent variables) clearly defined, valid, reliable, and implemented consistently across all study participants?	10. Was the exposure(s) assessed more than once over time?	11. Were the outcome measures (dependent variables) clearly defi ned, valid, reliable, and implemented consistently across all study participants?	12. Were the outcome assessors blinded to the exposure status of participants?	13. Was loss to follow-up after baseline 20% or less?	14. Were key potential confounding variables measured and adjusted statistically for their impact on the relationship between exposure(s) and outcome(s)?	total scores	
	Yes/No/Not reported (NR) or cannot determine (CD) or not applicable (NA)	Yes/No/Not reported (NR) or cannot determine (CD) or not applicable (NA)	Yes/No/Not reported (NR) or cannot determine (CD) or not applicable (NA)	Yes/No/Not reported (NR) or cannot determine (CD) or not applicable (NA)	Yes/No/Not reported (NR) or cannot determine (CD) or not applicable (NA)	Yes/No/Not reported (NR) or cannot determine (CD) or not applicable (NA)	Yes/No/Not reported (NR) or cannot determine (CD) or not applicable (NA)	Yes/No/Not reported (NR) or cannot determine (CD) or not applicable (NA)	Yes/No/Not reported (NR) or cannot determine (CD) or not applicable (NA)	Yes/No/Not reported (NR) or cannot determine (CD) or not applicable (NA)	Yes/No/Not reported (NR) or cannot determine (CD) or not applicable (NA)	Yes/No/Not reported (NR) or cannot determine (CD) or not applicable (NA)	Yes/No/Not reported (NR) or cannot determine (CD) or not applicable (NA)	Yes/No/Not reported (NR) or cannot determine (CD) or not applicable (NA)		
Canetti et al. (2018) [24]	Yes	Yes	Yes	Yes	Yes	Yes	Yes	NA	NA	NA	Yes	NR	NR	NR	8	Fair
Clement et al. (2020) [32]	Yes	Yes	Yes	Yes	No	Yes	Yes	NA	NA	NA	Yes	NR	Yes	Yes	9.5	Fair
Crizer et al. (2021) [28]	Yes	Yes	NR	Yes	No	Yes	Yes	NA	NA	NA	Yes	NR	Yes	Yes	8.5	Fair
Foissey et al. (2023) [26]	Yes	Yes	NR	Yes	No	Yes	Yes	NA	NA	NA	Yes	No	Yes	NR	8	Fair
Negrín et al. (2020) [31]	Yes	Yes	NR	Yes	Yes	No	No	NA	NA	NA	Yes	NR	Yes	Yes	7.5	Fair
Negrín et al. (2021) [2]	Yes	Yes	NR	Yes	No	No	Yes	NA	NA	NA	Yes	Yes	Yes	NR	8	Fair
Park et al. (2019) [33]	Yes	Yes	NR	Yes	No	No	Yes	NA	NA	NA	Yes	NR	Yes	Yes	8	Fair
Wong et al. (2019) [25]	Yes	Yes	Yes	Yes	Yes	Yes	Yes	NA	NA	NA	Yes	NR	NR	NR	8	Fair
Wu et al. (2021) [27]	Yes	Yes	No	Yes	No	Yes	Yes	NA	NA	NA	Yes	NR	Yes	NR	8	Fair

Outcomes

**Figure 3:**
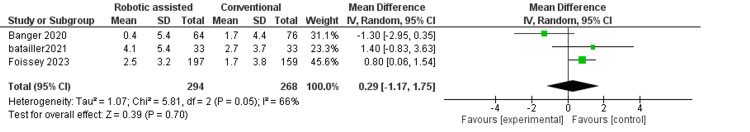
Forest plot of the change in hip-knee-ankle angle (HKA score).

Change in hip-knee-ankle angle: This outcome measures the change in the alignment of the hip, knee, and ankle after surgery. We found that robotic-assisted surgery had a better alignment than conventional surgery (MD = 0.86, 95% CI = 0.16-1.56, p = 0.02) (Figure 3). The results were consistent across the studies (p = 0.62, I^2^ = 0%).

**Figure 4:**
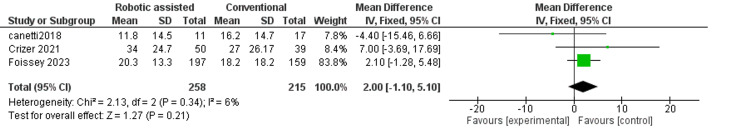
Forest plot of the International Knee Score.

Change in the International Knee Society score: This outcome measures the change in the function and pain of the knee after surgery. We found no difference between robotic-assisted and conventional surgery (MD = 2.00, 95% CI = -1.10-5.10, p = 0.21) (Figure 4). The results were consistent across the studies (p = 0.34, I^2^ = 6%).

**Figure 5:**
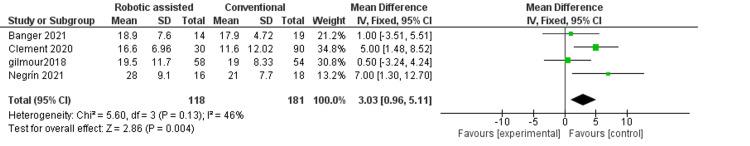
Forest plot of Oxford knee score.

Change in Oxford knee score: This outcome measures the change in the quality of life and satisfaction of the patient two to five years after surgery. We found that robotic-assisted surgery had a better quality of life and satisfaction than conventional surgery (MD = 3.03, 95% CI = 0.96-5.110, p = 0.004) (Figure 5). The results were moderately consistent across the studies (p = 0.13, I^2^ = 46%).

**Figure 6:**
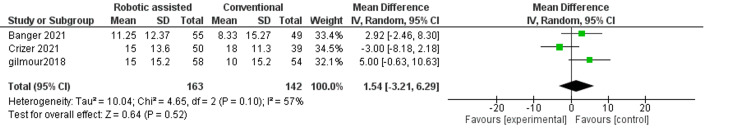
Forest plot of change in the range of motion.

Change in range of motion: This outcome measures the change in the movement of the knee after surgery. We found no difference between robotic-assisted and conventional surgery (MD = 1.54, 95% CI = -3.21-6.29, p = 0.52) (Figure 6). The results were inconsistent across the studies (p = 0.10, I^2^ = 57%).

**Figure 7:**
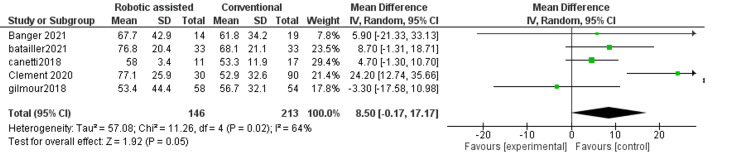
Forest plot of Forgotten joint score.

Forgotten joint score: This outcome measures the change in the awareness of the artificial joint after surgery. We found no difference between robotic-assisted and conventional surgery (MD = 4.75, 95% CI = -0.02-9.52, p = 0.05) (Figure 7). The results were consistent across the studies (p = 0.61, I^2^ = 0%).

**Figure 8:**
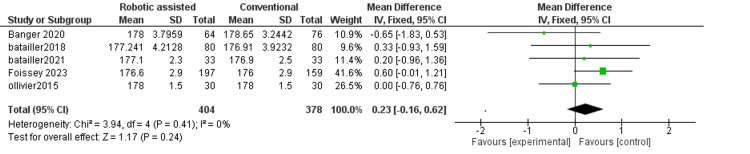
Forest plot of hip-knee-ankle angle (HKA score) postoperatively.

Hip-knee-ankle angle postoperatively: This outcome measures the alignment of the hip, knee, and ankle after surgery. We found no difference between robotic-assisted and conventional surgery (MD = 0.23, 95% CI = -0.16-0.62, p = 0.24) (Figure 8). The results were consistent across the studies (p = 0.41, I^2^ = 0%).

**Figure 9:**
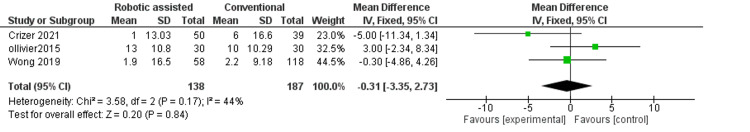
Forest plot of mental health.

Health status: This outcome measures the mental and physical health of the patient after surgery. Regarding mental health, we found no difference between robotic-assisted and conventional surgery (MD = -0.31, 95% CI = -3.35-2.73, p = 0.84) (Figure 9). The results were moderately consistent across the studies (p = 0.17, I^2^ = 44%). 

**Figure 10:**
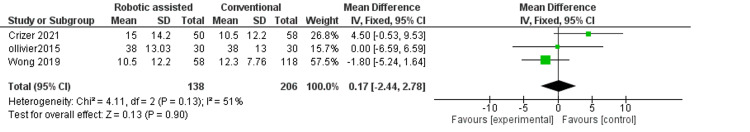
Forest plot of physical health.

Regarding physical health, we found no difference between robotic-assisted and conventional surgery (MD = 0.17, 95% CI = -2.44-2.78, p = 0.90) (Figure 10). The results were inconsistent across the studies (p = 0.13, I^2^ = 51%).

**Figure 11:**
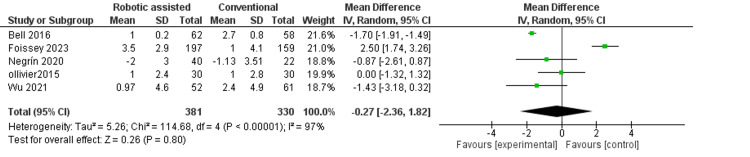
Forest plot of tibial slope.

Tibial slope: This outcome measures the angle of the tibia bone after surgery. We found no difference between robotic-assisted and conventional surgery (MD = -0.27, 95% CI = -2.36-1.82, p = 0.80) (Figure 11). The results were very inconsistent across the studies (p < 0.01, I^2^ = 97%).

**Figure 12:**
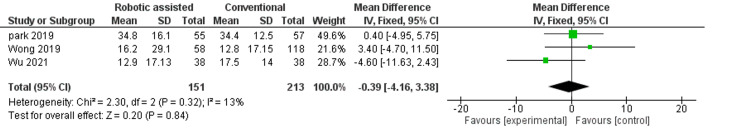
Forest plot of Western Ontario and McMaster Universities Osteoarthritis (WOMAC) pain score.

WOMAC pain score: This outcome measures the pain level of the patient after surgery the WOMAC questionnaire. We found no difference between robotic-assisted and conventional surgery (MD = -0.39, 95% CI = -4.17-3.38, p = 0.84) (Figure 12).

Discussion

We conducted a meta-analysis of 16 studies that compared the outcomes of two types of UKA, which are surgeries that replace only one part of the knee joint. One type was robotic-assisted surgery, which uses a computer-controlled device to assist the surgeon, and the other type was conventional surgery, which does not use any robotic device. We compared various outcomes that measured the alignment, function, pain, and quality of life of the patients after surgery. We found that robotic-assisted surgery had better outcomes than conventional surgery in two aspects, namely, hip-knee ankle angle, which measured how well the hip, knee, and ankle were aligned after surgery, and Oxford knee score, which measured how satisfied and happy the patients were with their surgery. However, we found no difference between robotic-assisted and conventional surgery in other aspects, such as IKS, which measured how well the knee functioned and how much pain the patients felt; the range of motion, which measured how much the patients could move their knee; forgotten joint score, which measured how much the patients were aware of their artificial joint; hip-knee ankle angle postoperatively, which measured the alignment of the hip, knee, and ankle after surgery; health status, which measured the mental and physical health of the patients; tibial slope, which measured the angle of the tibia bone after surgery; and WOMAC pain score, which measured the pain level of the patients using a questionnaire.

The hip-knee-ankle angle was significantly lower in the robotic-assisted group compared to the conventional unicompartmental in three studies (p = 0.02). These results match those of another systematic review and a meta-analysis conducted by Ren et al. which also indicated that the robotic-assisted group had a lower hip-knee-ankle angle (p = 0.04). No significant change was noted in the range of motion between the two surgical approaches when it came to the change of motion which matched the results of this meta-analysis [35].

Regarding the WOMAC pain scale, our analysis showed no significant difference when comparing the two surgical approaches, which complies with a systematic review and a meta-analysis conducted by Karunaratne et al. of four studies [36]. Although our study showed no statistical difference between conventional UKA and the robotic-assisted approach in terms of the forgotten joint score, another meta-analysis indicated a statistical difference between the two approaches when comparing forgotten joint scores [37].

A meta-analysis conducted by Zhang et al. which included 11 studies indicated no significant difference between robotic-assisted and conventional UKA in terms of forgotten joint score, Oxford knee score, and range of motion, which matches the results of this study [38]. Regarding the tibial slope, our analysis showed no significant difference between the two surgical approaches, although another systematic review by Robinson et al. showed that based on two studies, the robotic-assisted approach had a significantly lower tibial slope when compared to the conventional UKA [39].

The use of robotic-assisted knee arthroplasty has increased in the past years due to evidence of improved quality of life and increased accuracy [40]. It has been shown to be associated with fewer revision surgeries and more cost savings [41], more normal knee motion, and better results when walking [42]. However, it was proven in a study by Hafar et al. that the robotic-assisted approach is associated with higher operative time, a higher energy expenditure per minute, and increased heart rate. Therefore the robotic-assisted approach can be associated with less favorable outcomes. On the other hand, Hafar et al. found that conventional approach is associated with higher surgeon neck flexion [43].

Furthermore, a study conducted in Ohio found no statistical difference when comparing the two approaches in average time in recovery, average postoperative hematocrit, and average postoperative hemoglobin [44]. Regarding the time required for physical therapy, the robotic-assisted approach required significantly less time [44,45]. Moreover, the robotic-assisted approach was found to be associated with less pain, less analgesia requirement, and less time to straight leg raise, with no difference in blood loss [45].

Finally, no difference was noted between the two approaches in terms of five-year survival analysis, as well as no difference in postoperative complications was recorded [46-48].

There are a few limitations to this study. First, some included studies were not RCTs which can increase the risk of bias. Second, the number of samples included for most outcomes was not sufficiently large. Future studies with a large sample size are needed to evaluate the infection rate between both groups.

## Conclusions

We analyzed 16 studies that compared two types of UKA, which are surgeries that replace only one part of the knee joint. One type used a robotic device to help the surgeon, and the other type did not. We compared different outcomes that measured how well the knee was aligned, how well it worked, how much pain the patients experienced, how healthy they were, and how much they noticed their artificial joints. We discovered that the type of surgery that used a robotic device had better outcomes in the hip-knee-ankle Angle, which measured the alignment of the hip, knee, and ankle, and Oxford knee score, which measured the function and satisfaction of the knee. However, we did not discover any difference in the other outcomes between the two types of surgery. Therefore, we suggest that the type of surgery that uses a robotic device may be better than the other type in some ways, but we need more research to confirm this and to determine how this affects the patients in the long run and how much it costs.
